# Comparison of Diagnostic Performance of Five Different Ultrasound TI-RADS Classification Guidelines for Thyroid Nodules

**DOI:** 10.3389/fonc.2020.598225

**Published:** 2020-11-16

**Authors:** Ruoning Yang, Xiuhe Zou, Hao Zeng, Yunuo Zhao, Xuelei Ma

**Affiliations:** ^1^ Department of Biotherapy, State Key Laboratory of Biotherapy, West China Hospital, Cancer Center, Sichuan University, Chengdu, China; ^2^ West China Hospital, West China School of Medicine, Sichuan University, Chengdu, China; ^3^ Department of Thyroid Surgery, West China Hospital of Sichuan University, Chengdu, China

**Keywords:** thyroid nodule, meta-analysis, TI-RADS, ultrasound, malignancy, diagnostic performance

## Abstract

**Objectives:**

We aimed to evaluate and compare the diagnostic performance of five ultrasound thyroid imaging reporting and data system (TI-RADS) classification guidelines for thyroid nodules through a review and meta-analysis.

**Methods:**

We searched for relevant studies before February 2020 in PubMed. Then we pooled the sensitivity, specificity, likelihood ratios, diagnostic odds ratios, and area under the summary receiver operating characteristic curves. And the diagnostic odds ratios were used to compare the performance.

**Results:**

We totally included 19 studies with 4,696 lesions in this research. The pooled sensitivity of American College of Radiology (ACR) guidelines, American Thyroid Association (ATA) guidelines, TI-RADS proposed by Kwak (Kwak TI-RADS), Korean Thyroid Association/Korean Society of Thyroid Radiology (KTA/KSThR) guidelines for malignancy risk and European Thyroid Association (ETA) guidelines is between 0.84 and 0.94. The pooled specificity is 0.68, 0.44, 0.62, 0.47, and 0.61, respectively. And the RDOR is 1.57 (ACR *vs* ATA), 1.37 (ACR *vs* ETA), 1.80 (ACR *vs* Kawk), 1.74 (ARC *vs* KTA).

**Conclusions:**

The results suggest that five classification guidelines are all effective methods for differential diagnosis of benign and malignant thyroid nodules and ACR guideline is a better choice.

## Introduction

Thyroid nodules are easily found in the general population, especially in women (1), and about 10% of patients with thyroid nodules are at risk of malignancy, and the percentage keeps going up (2, 3). Malignant nodules and benign nodules are treated in completely different ways. It’s still a big challenge for clinicians to rule out malignancy of the thyroid nodules. At present, ultrasound is a primary, cheap, noninvasive, fast, and valuable tool to identify the thyroid nodules. For suspected thyroid nodules, a surgery or fine-needle aspiration cytology (FNAC) is recommended (4). Benign and malignant nodules have some similar ultrasound features from modulation to size. The ultrasound diagnosis varies with the experience of radiologists, and operators, image acquisition and interpretation are subjective which can easily lead to misdiagnosis or overtreatment (5).

To conduct an objective detection, the thyroid imaging reporting and data system (TI-RADS) was proposed, which is used to classify thyroid nodules and recommend further treatment (6). Nowadays, there are five common classification systems used in clinic. Among the guidelines, the American College of Radiology (ACR) guidelines, the Korean Thyroid Association/Korean Society of Thyroid Radiology (KTA/KSThR) guidelines, and the European Thyroid Association (ETA) guidelines are recommended by the radiological association, and the American Thyroid Association (ATA) guidelines are in clinical guidelines (1, 7–9).

Although these five guidelines prove to be effective in managing thyroid nodules, there are no guidelines based on a lot of reliable data to prove which is the best (10). And many clinical trials in progress are used to compare their effectiveness, but these results are biased. The primary purpose of this research is to compare the diagnostic effectivity of the five guidelines for thyroid nodules to address the lack of consistency and avoid wasting of medical resources.

## Method

### Literature Search Strategy

We followed the guidelines for the systematic review and meta-analysis of diagnostic studies. Then we too retrieved PubMed for related studies with English language only before February 2020, using the terms as follows: “sensitivity”, “specificity”, “TI-RADS (or thyroid imaging reporting and data system)”, “ACR (or The American Thyroid Association)”, “ATA (or American Thyroid Association)”, “Kwak (or TI-RADS proposed by Kwak)”, “ETA (or EU TI-RADS)”, “KTA (or Korean Thyroid Association/Korean Society of Thyroid Radiology)”. Two reviewers (RN Yang and YN Zhao) independently reviewed the articles in accordance with the inclusion and exclusion criteria. Disagreements were adjusted by consensus (XL Ma).

### Inclusion and Exclusion Criteria

Studies with following inclusion criteria were included: (a) There is enough general information in the article. (b) One or more guidelines are used to evaluate the ultrasound features of thyroid nodules. (c) The study has definite diagnostic criteria. (d) There is sufficient data in the article, whether it is data that can be found directly in the article (sensitivity, specificity, and PPV) or data that can be calculated based on the article [positives (TP), true negatives (TN), false positives (FP), and false negatives (FN)] to fill the diagnostic 2 × 2 table (FN, FP, TP, and TN). And the exclusion criterion is that data in the article is not enough or the grading system is not designed to evaluate ultrasound features. Finally, a total of 19 articles are included.

### Data Extraction

Two reviewers (RN Yang and YN Zhao) picked up some main characters from the studies as following: author, year, country, number of patients, number of nodules, mean age, involved guideline, gold standard, malignant lesions, and benign lesions. And we obtained the four numbers of TP, TN, FP, and FN for each guideline in different studies by two ways: (1) We got the data from the article directly. (2) Based on the data (sensitivity, specificity, PPV, and NPV) obtained from the articles, we finished the diagnostic 2 × 2 table. CAL software was use here (11).

### Statistical Analysis

On the bases of TP, TN, FP, and FN, we computed the pooled sensitivity, specificity, positive and negative likelihood ratios (PLR and NLR), and diagnostic odds ratio (AUC), with 95% confidence intervals (CI), using the Meta-Disc version 1.4 statistical software (12).

Additionally, using the Meta-Disc version 1.4 statistical software (12), we examined the relationship between sensitivity and specificity by constructing the summary receiver operator characteristic (SROC) curves (13).

At last, we made a head-to-head comparison using R 3.5.1 to calculate the relative diagnostic odds ratio (RDOR) with 95% CI. According to the RDOR, we compared the diagnostic performance among the five guidelines. At comparison, classified into A and B, two guidelines were involved. In A *vs* B, when the value is greater than 1, A has higher performance. If the value is smaller than 1, B has greater performance. When the value is greater, the performance is better. For all studies, the inconsistency index (I^2^) and χ^2^ test were used to assess heterogeneity, and it was considered high heterogeneity if the I^2^ value was higher than 50% (14). A random-effect model was chosen in this research (15).

### Quality of Studies and Publication Bias

We used Quality Assessment of Diagnostic Accuracy Studies version 2 (QUADAS-2) performed in Review Manager 5.2 to assess the quality of the studies included in this analysis. The method mainly evaluated the articles from four domains: (a) patient selection, (b) index test, (c) reference standard, and (d) flow and timing (16). Each domain is rated as three risks (low, high, and unclear). Publication bias was evaluated by the funnel plot asymmetry test using Stata version 11.0 software.

## Results

### Literature Research and Study Characteristics

**Figure 1:**
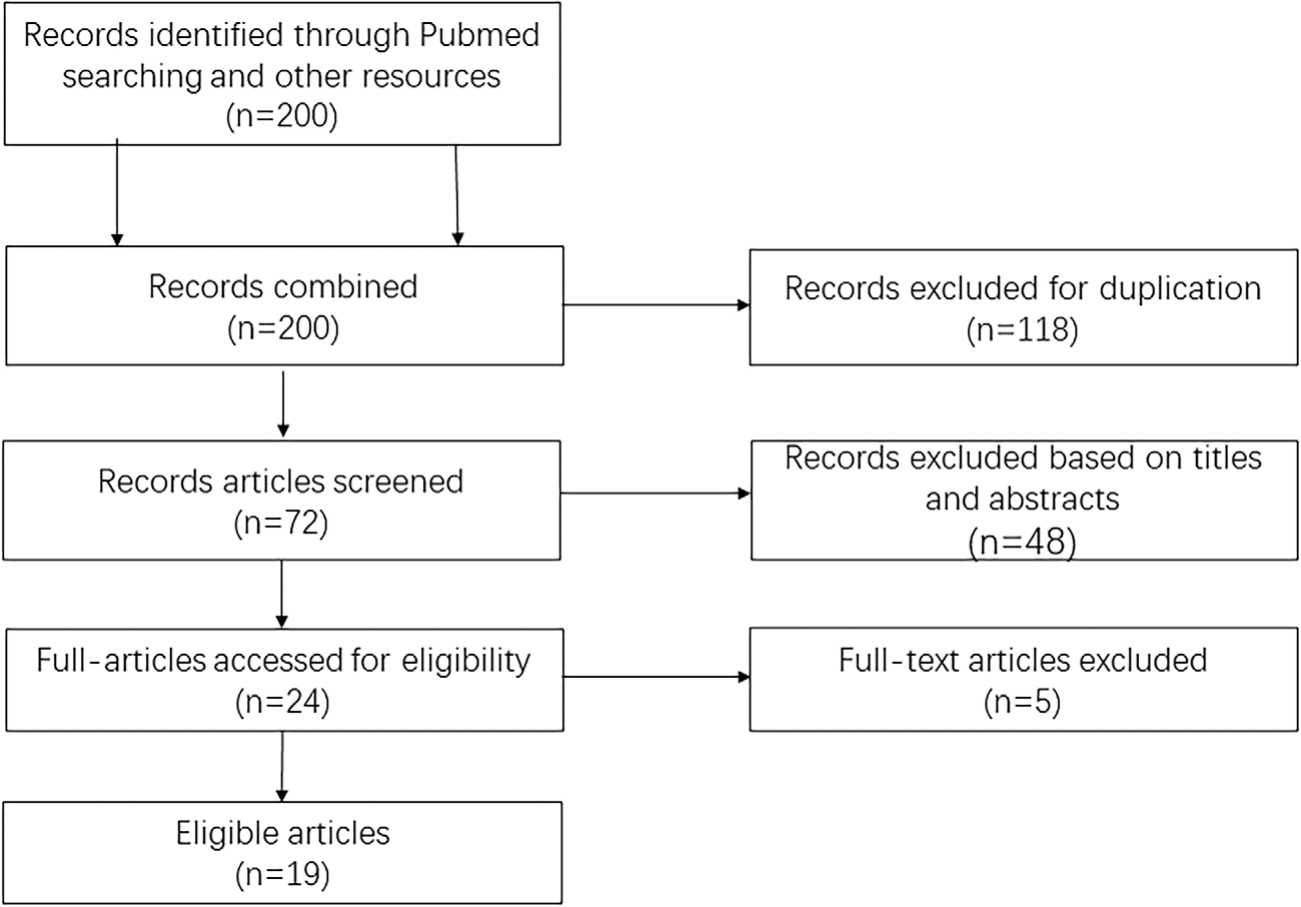
Flowchart of the literature search and selection schema.

At first, we searched 200 articles by reading their abstract, and 166 articles didn’t agree with the inclusion criterion. Then we reviewed the rest of the articles further, and 11 articles didn’t have enough data to finish the 2 × 2 table. Another five articles were not related to ultrasound features. Therefore, 19 articles (17–35) were included in this study. The process of including articles is in **Figure 1**.

The studies were published from 2015 to 2020.The number of patients is from 92 to 4,585, and the number of nodules in the included articles varies from 100 to 4,696, which means some patients have more than one nodule. All data were calculated based on the number of nodules. All thyroid nodules were diagnosed of malignancy through postoperative pathological results or the pathology results of FNAC. We totally included 19 articles, 12 of which involved ACR TI-RADS. 10 articles involved ACR guidelines, and Kwak TI-RADS was mentioned in six articles. The data of the KTA guideline and EU TI-RADS were obtained from four articles respectively.

The above characteristics were shown in **Table 1**.

**Table 1:** Baseline characteristics of included studies.

Author	Year	Country	Patients, n	Nodules, n	MeanAge	Guidelines	Standard	Malignant lesions	Benign lesions
Zhang (18)	2020	China	1,271	1,271	48	ACR/ATA/Kwak/KTA	Needle biopsy, surgical resection	736	535
Gao (19)	2019	China	1,764	2,544	—	ACR/ATA/Kwak	Surgical resection	1,681	863
Barbosa (20)	2019	Brazil	139	140	49	ACR/ATA	Needle biopsy, surgical resection	66	74
Jabar (21)	2019	India	127	127	—	ACR/Kwak	Needle biopsy, surgical resection	23	104
Xv (24)	2019	China	370	432	43	ACR	Needle biopsy, surgical resection	258	174
Yoon (25)	2019	Korea	1,836	2,274	55	ACR/KTA	Needle biopsy, surgical resection	300	1,974
Huang (26)	2019	USA	137	250	58	ACR/ATA	Surgical resection	65	185
Ruan (27)	2019	China	918	1,001	46	ATA	Needle biopsy, surgical resection	392	609
Wang (22)	2017	China	1,011	1,011	51	Kwak	Surgical resection	464	547
Liu (23)	2015	China	2,921	3,980	52	Kwak	Needle biopsy	228	3,752
Ha (17)	2018	Korea	1,802	2,000	51	KTA, ATA, ACR	Needle biopsy, surgical resection	1,546	454
Mohammadi (28)	2019	Canada	—	425	—	ATA	Needle biopsy	31	394
Wu (29)	2019	China	894	1,000	—	ATA, ACR	Needle biopsy, surgical resection	530	470
Xu (30)	2019	China	2,031	2,465	48	KTA, ACR, ETA	Surgical resection	885	1,146
Yoon.J (31)	2017	Korea	4,585	4,696	51	ATA, Kwak	Needle biopsy, surgical resection	1,044	3,652
Hoang (32)	2018	USA	92	100	52	ACR	Needle biopsy, surgical resection	15	85
Li (33)	2019	China	128	130	48	ACR	Needle biopsy, surgical resection	73	57
Trimboli (34)	2019	Switzerland	475	1,058	53	ETA	Needle biopsy, surgical resection	—	—
Maino (35)	2018	Italy	340	432	57	ATA. ETA	Needle biopsy	—	—

### Diagnostic Accuracy

After pooling all the data of the 19 studies together, we got the final data. The pooled sensitivity of ACR guidelines, ATA guidelines, Kwak TI-RADS, KTA guidelines for malignancy risk, and ETA guidelines is between 0.84 and 0.94. The pooled specificity is 0.68, 0.44, 0.62, 0.47, and 0.61, respectively. We also build SROC curves showing the area under the curve (AUC) with 0.8553, 0.9101, 0.8976, 0.9022, and 0.8810, respectively, for ACR, Kwak TI-RADS, ATA, KTA, and EU TI-RADS guideline groups, on behalf of the accuracy. All pooled sensitivity, specificity, PLR, NLR, DOR, and the AUC values for all the reference standards are shown in detail in **Table 2**. As for RDOR, we found a high result when ACR was compared with other guidelines. The specific results are listed in **Table 3**.

**Table 2:** Pooled estimates of the sensitivity, specificity, PLR, NLR, DOR, AUC, and SE (AUC).

Reference guideline	N^a^	Pooled sensitivity (95% CI)	Pooled specificity (95% CI)	Pooled PLR (95% CI)	Pooled NLR (95% CI)	Pooled DOR (95% CI)	AUC	SE (AUC)
ACR	13	0.85(0.84–0.86)	0.68(0.6–0.69)	2.98(2.37–3.75)	0.22(0.16–0.29)	15.23(9.23-25.11)	0.8553	0.0311
Kawk	6	0.94(0.94–0.95)	0.62(0.6–0.63)	3.23(0.90–11.61)	0.08(0.04–0.16)	43.15(19.09–97.53)	0.9101	0.0621
ATA	10	0.94(0–94-0.95)	0.44(0.43–0.45)	2.06(1.54–2.75)	0.16(0.10–0.28)	13.33(5.90-30.14)	0.8976	0.0414
KTA	4	0.85(0.83-0.86)	0.47(0.46-0.48)	2.60(1.2–5.57)	0.18(0.08-0.39)	14.57(5.77-36.84)	0.9022	0.0430
ETA	4	0.85(0.83-0.87)	0.61(0.59-0.62)	2.84(1.43-5.64)	0.21(0.13-0.34)	13.18(4.89-35.5)	0.8810	0.0561

na, number of studies; PLR, positive likelihood ratios; NLR, negative likelihood ratios; DOR, diagnostic odds ratio; AUC, area under the curve.

**Table 3:** Relative diagnostic odds ratio (RDOR) with 95% confidence limit.

B A	ACR	ATA	ETA	Kawk	KTA
ACR	–	0.6387(0.3678–1.1090)	0.7308(0.3000–1.7803)	0.5564(0.2552–1.2131)	0.5734(0.2759–1.1919)
ATA	1.5658(0.9017–2.7189)	–	1.1443(0.4532–2.8897)	0.8713(0.3995–1.8999)	0.8979(0.4072–1.9802)
ETA	1.3683(0.5617–3.3332)	0.8739(0.3461–2.2067)	–	0.7614(0.2498–2.3208)	0.7846(0.3075–2.0020)
Kawk	1.7972(0.8243–3.9183)	1.3134(0.5264–2.5028)	1.3138(0.4309–4.0035)	–	1.0306(0.3927–2.7048)
KTA	1.7439(0.8390–3.6247)	1.1137(0.5050–2.4561)	1.2745(0.4995–3.2518)	0.9703(0.3697–2.5466)	–

SROC, summary receiver operator characteristics.

### Quality Assessment

**Figure 2:**
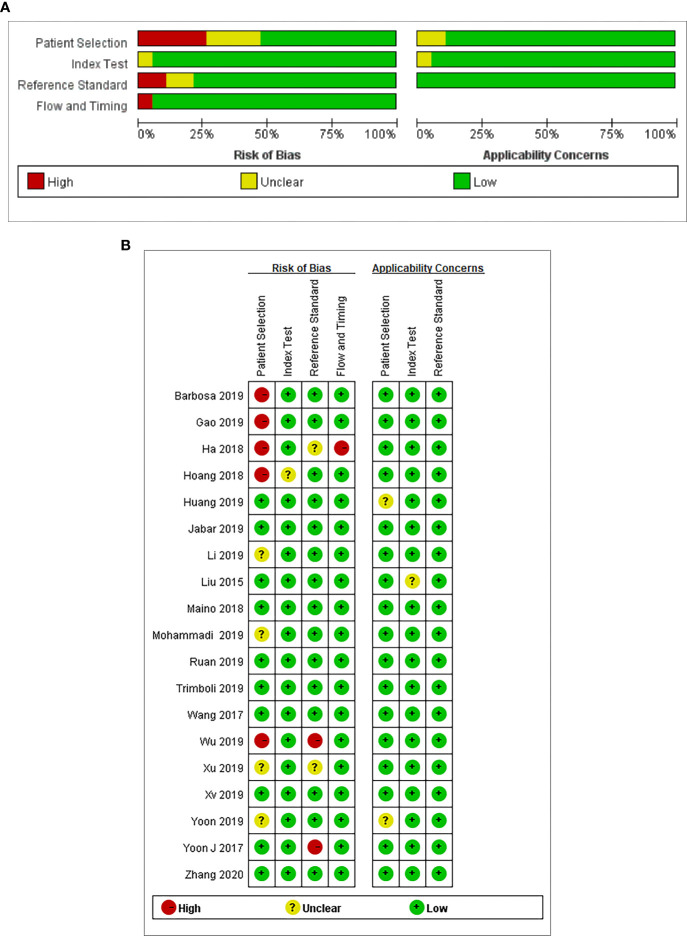
**(A)** Risk of bias and applicability concerns graph: review authors’ judgments about each domain presented as percentages across the included studies. **(B)** Risk of bias and applicability concerns summary: review authors’ judgments about each domain for each included study.

The results of the quality assessment are outlined in **Figure 2**. In conclusion, the quality of the studies was satisfactory.

### Assessment of Publication Bias

There is no clear publication bias for DOR of the five guidelines.

## Discussion

TI-RADS classification guidelines classify the thyroid nodules according to the imaging characteristics under ultrasound, including the size, number, calcification, boundary, echoic pattern, aspect ratio, and internal structure. The guidelines are aimed to help determine which thyroid nodules require FNAC to reduce overdiagnosis or missed-diagnosis. The reduction of unnecessary FNAC can prevent the waste of economy and the physical pain of patients. It can also guide further treatment and estimate the risk of recurrence. However, the recommended size thresholds for FNAC are different in different guidelines. At present, there are many studies about the diagnostic efficacy of the five guidelines, but the results vary. These differences between studies may be due in part to differences among observers and study populations, especially in retrospective studies. In this research, we included 19 studies to analyze the diagnostic efficacy of the five diagnostic criteria.

Our meta-analysis systematically estimated the diagnostic efficacy of five different ultrasound classification guidelines in detecting malignancy risk. The pooled sensitivity of the ACR TI-RADS, ATA guidelines, Kwak TI-RADS, KTA guidelines, and ETA is between 0.84 and 0.94. The pooled specificity is 0.68, 0.44, 0.62, 0.47, and 0.61, respectively. The AUC which can represent the diagnostic performance of the ACR TI-RADS, ATA guidelines, Kwak TI-RADS KTA guideline, and ETA is 0.8553, 0.8976, 0.9101, 0.9022, and 0.8810. In theory, AUC above 0.8 is diagnostic (36). The results of our research suggested that all the five guidelines have property. Besides, ACR guidelines showed the best diagnostic performance in the head to head comparison.

Our results were similar with a previous meta-analysis published in 2019 (37). But that article just included 12 studies with 18,750 thyroid nodules, and the data it included was used to describe the unnecessary FNA rates. There are 19 articles of 24,325 thyroid nodules in our research. Compared with the published article, we can include the articles with indirect data and finish the diagnostic 2 × 2 table using Cal software. Besides, in an article we both included, our article included the data of diagnostic performance for malignant thyroid nodules which better describes the diagnostic efficiency. It can influence the pooled results.

The TI-RADS guidelines based on ultrasound have been widely used in clinics, providing recommendation for further diagnosis and treatment while reducing the influence of subjective factors in diagnosing. In our study, we can see the five guidelines all have great diagnostic performance with high AUC above 0.8. However, there are similarities and differences among the five guidelines in structure, risk stratification, size thresholds, and diagnostic performance. More studies need to be done. The structure of the five classification guidelines is internally different. The ACR guidelines and Kwak TI-RADS are point-based systems, and the other three guidelines are based on the pattern. Compared with point-based guidelines, the simplified pattern-based guidelines are more intuitive and feasible clinically but with decreased accuracy. Although the point-based guidelines are cumbersome, they’re easy to control by clinical doctors, especially estimating individual nodules, which are with great accuracy. However, in clinical application, complex analyses and calculations always require the help of computers (10, 38). Every guideline has been divided into several categories to evaluate the thyroid nodules. As the risk stratification categories rise, the risk of the malignancy is increased, but the five guidelines have differences in the classification. For example, a category five or four thyroid nodule in ETA may be classified as ACR T4/3 or KTA T4/3, and a nodule of KTA T3 (low suspicion) and ETA category 3 (low risk) may be classified as ACR T2, which means not suspicious. Different classification criteria like the above may lead to different specificities, and as the results in our research, the ACR guidelines surely had the highest specificity. It also means less recommendation for FNAC, but the rate of misdiagnosis increases. We need more studies to discuss. As for the performance of recommendation for FNAC, the five guidelines have different size thresholds, and the thresholds also change with categories in different guidelines. For example, for ACR TI-RADS, the threshold of categories three, four, and five is 2.5, 1.5, and 1cm, respectively (39). Some studies (17) have shown ACR TI-RADS have the most effective criteria which can avoid the unnecessary biopsies effectively. Our results also confirmed this, with the highest RDOR for ACR TI-RADS. Nodule size is an important standard for guidelines and further treatment. The too large thyroid nodules with low malignancy risk will suggest surgery or FNAC.

In addition, there are several limitations in this research. Firstly, the final diagnosis was determined by cytology or pathology. It may be influenced by the operators or observers, with possible bias. Especially in retrospective studies, we are not sure whether subjective factors affect the diagnosis. This influence can’t be avoided. The second limitation is caused by the patient selection of included studies. Some studies have included more patients with malignant nodules which could influence the sensitivity and specificity. Thirdly, we didn’t have enough data for KTA guidelines and ETA to analyze. Lastly, all analyses are based on the ultrasound; the intra-observer and inter-observer variability still exists.

In conclusion, our research indicates that the five classification guidelines are all effective methods for differential diagnosis of benign and malignant thyroid nodules. They can be used before further diagnosis or treatment as an effective recommendation. In head to head comparison, the result suggests ACR guideline is a better choice in the benign and malignant diagnosis with high diagnostic accuracy. However, we still need more studies to prove our findings.

## Author Contributions

All authors directly participated in the planning, execution, or analysis of the study and wrote the manuscript. RY conducted the literature review, planned and performed all statistical analyses. HZ and XZ provided input and direction for the analytic strategy and editing of the manuscript. YZ reviewed the included articles and provided editing of the manuscript. XM provided technical quality control to ensure accuracy of reported results. All authors contributed to the article and approved the submitted version.

## Conflict of Interest

The authors declare that the research was conducted in the absence of any commercial or financial relationships that could be construed as a potential conflict of interest.
